# Inside or Outside? A Qualitative Comparative Study of 2 Recovery Colleges in Sweden and Norway

**DOI:** 10.2196/91076

**Published:** 2026-08-11

**Authors:** Lina Al-Adili, Ragnhild Jensen Roaldseth, Henning Pettersen, Mats Brommels

**Affiliations:** 1 Medical Management Centre, Department of Learning, Informatics, Management and Ethics, Karolinska Institutet, Tomtebodavagen 18 A, Stockholm, 17177, Sweden, 46 0705526930; 2 Faculty of Health and Social Sciences, University of Inland Norway, Elverum, Innlandet, Norway

**Keywords:** recovery, recovery college, mental health, self-management, patient-centeredness, person-centeredness, patient-staff collaboration, co-design, coproduction

## Abstract

**Background:**

Recovery colleges (RCs) provide users of mental health services with self-management and coping skills, along with actions to promote social inclusion and reduce stigma. Research shows that recovery principles in mental health have the potential to improve services, but are poorly implemented. Better knowledge and understanding of RCs among mental health staff might help in addressing this challenge.

**Objective:**

In order to shed light on that assumption, the study compares 2 RCs that differ in their formal relationship with the mental health service—1 “embedded” and 1 “freestanding.” This comparative study aims to uncover whether there are differences in the perceived value of the RCs by participants, and in mental health staff views on the benefits and challenges related to RCs, and the potential for collaboration.

**Methods:**

The design is an explanatory comparative case study. Thick descriptions of the cases were based on document analysis and stakeholder interviews, subjected to content analysis. A “strategic change management model” guided data collection and analysis. Empirical patterns demonstrating the character and interrelations of the context, content, process, and outcome were identified in the case descriptions, forming tentative explanations of benefits and challenges observed.

**Results:**

RC participants reported some differences in experiences possible to relate to course content. Information on mental health services enabled a more efficient use of those resources. A focus on communication and coping strategies helped in building bonds and relationships and finding support in social networks. Both RCs supported empowerment and reduced the sense of stigma. Mental health professionals had a positive image of the RCs but were not well informed about their activities. Few saw the college as an activity that could complement their professional services but had interesting reflections on their workplaces in relation to recovery. Exposure to an RC could support a reorientation from “diagnosis-centered” to a more patient-centered care culture. Users offering peer support could be “go-betweens” and “bridge builders” to mental health services.

**Conclusions:**

Mental health staff aware of RCs and having knowledge of their benefits will provide support and guide users to participate. Such positive experiences are more important as a promoter of collaboration with users and the interest to contribute to a recovery-oriented care culture than the organizational form of the RC and its formal link to mental health services.

## Introduction

### Background

Persons in need of psychiatric treatment and care have been front-runners in terms of self-management of disease and taking control over their lives despite disease, as well as organizing peer support. This started in the United States as a consumer movement in the 1970s [1] and coincided with the deinstitutionalization of psychiatric services. At the same time, longitudinal studies of patients with mental disorders changed the previously pessimistic view on severe psychiatric disorders, well in line with the personal experience of people that they were, despite a psychiatric diagnosis, able to lead meaningful and productive lives. Those studies also showed that the capacity of patients to recover fully or learn to manage their condition with strategies which were in many instances developed outside formal treatment settings [1,2]. During the last 30 years, an emergence of locally based services, therapeutic societies, and the development of social psychiatry have taken place, which has generated both new knowledge and new practices [3,4].

Perkins et al [5] contrast this patient-driven self-management activity from professional psychiatric care by referring to it as an “educational” rather than “therapeutic approach[es].” Instead of focusing on problems and dysfunctions, and limiting activities to therapies, the recovery movement supports people to identify and develop their talent and skills, explore their possibilities, and focus on achieving ambitions and goals. It has, consequently, also been defined as an “assets-based” approach, aiming at developing the “recovery capital” of patients, defined as “the array of social, psychological and cultural networks beyond professional inputs” [6]. It has subsequently been seen as complementary to specialist mental health services [7].

Recovery colleges (RCs) are examples of that educational approach. The first RC was established in Boston in the 1990s, initially named Recovery Learning Center. In Europe, the first RC was established in London in 2009, and today a total of 80 RCs are spread across Great Britain. Twenty other countries have followed suit [5]. An RC can best be characterized as an educational center based in the community with the overall aim to promote recovery among persons with mental health challenges by focusing on education and support [8,9]. These colleges organize, led by patient peers, courses in, inter alia, “Living with a mental disorder” and provide participants with skills and employment training, along with actions to promote social inclusion and reduce stigma. The courses are developed and co-designed, coproduced, and cofacilitated by both mental health professionals and persons with user experience.

Recovery is said to have its root in psychiatric rehabilitation, but its proponents tend to distance themselves from mainstream psychiatry [10]. On the other hand, initiatives such as UK RCs aim at moving beyond patient involvement to coproduction and the development of coproduced patient pathways. Importantly, Topor et al [11] emphasize that the lack of “recovery capital” among patients is associated with lack of trust in oneself, and that professionals are able to support if they gain acceptance as trustful agents by cocreating changes in those patients’ lives. They also report that professionals were concerned with the fragmentation and societal shortcomings of their care organizations [11].

Recent research shows that RCs have overall positive outcomes from their activities. Several studies on students’ experiences underscore that RCs provide a safe space and meaningful connections and give room for personal growth, empowerment, and hope [10,12-14]. Students using RCs seem to be, in general, representative of mental health service users, but some groups are underrepresented, such as men, older adults, ethnic minority groups, and those with developmental disorders [15,16]. RCs display consistent values and aims in supporting recovery but are diverse in their operations [8,17]. Furthermore, RCs differ widely in organizational structure and whether they are affiliated with a health trust or a local community. Research examining RC characteristics concerning their affiliation is sparse, but a recent study comprising 36 RCs across England found that those 2 RC types did not differ significantly on either clinical or any health service use outcomes [18].

The recovery movement within mental health is a prominent example of a patient-led activity that can improve the well-being of patients and, when carried out in cooperation with mental health services, contribute to their development into more patient- and person-centered organizations. Research shows that recovery principles in mental health have the potential to improve services, but that these principles are poorly implemented [19-21]. The challenge is to make the potential partnership come true by building trust, overcoming ideological barriers, and managing resistance to change among mental health professionals and their organizations.

These are organizational and leadership issues. However, the literature on RCs has until recently been scarce on organizational studies. The English RECOLLECT project, studying primarily RC effectiveness and cost-effectiveness, set out to identify organizational influences on fidelity and improvements in mental health outcomes and found that RCs basically fall into 2 categories: those located within National Health Service (NHS) Trusts with structured recovery planning and those outside the NHS that focused on social connection and community integration. No significant differences were found between those RC types in terms of perceived participant benefit [16]. Thaki et al [18] reported on how organizational factors facilitate or hinder the establishment and management of RCs. They identified the main organizational affiliation, roughly two-thirds of RCs being affiliated with a statutory health service (usually an NHS Trust) and one-third with a voluntary organization. Managers interviewed mentioned that the benefits of being affiliated with an NHS Trust were access to qualified professionals who can participate in coproducing RC workshops, higher professional staff awareness, and increased visibility to secondary service providers supporting service users, one example being employment agencies. Negative consequences were competition for resources within the NHS Trust, lack of support to the RC as higher priority is given to clinical services, and NHS bureaucratic rules restricting autonomy for RC operators. However, these insights were provided by an analysis of all interviews without identifying possible differences in experience between managers representing different organizational forms of RCs [18].

We address this gap in knowledge by analyzing and comparing 2 RCs in 2 different countries that differ in their formal relationship with the mental health service. One has a narrow focus and is embedded in a psychiatry organization engaging salaried staff with lived experience and the other is freestanding with voluntary workers and a wide range of activities. We chose those cases as we had since several years had access to the sites and had previously performed studies on the experience of participants. Despite the study on RCs aligned either to a mental health service or to the third sector [18], to our knowledge, no study involving an RC within psychiatry has been previously reported.

An educational program, called “the Patient School” and resembling an RC, is offered at the Region Stockholm (Sweden) Psychiatry Organisation by “user-influence coordinators” (UICs) and “staff with user experience” (SUEs) employed by the organization.

Sagatun Recovery (SR) (user-led center) in Hamar, Norway, is a freestanding facility organizing a range of recovery-supporting activities, locally, regionally, and nationally, with the overall aim to promote user involvement, empowerment, and recovery. Its “self-empowerment course (SEC)” is organized as an RC.

### Purpose and Aims

The purpose of this study is to make a systematic comparison of the embedded versus the freestanding RC in terms of their perceived value and the views of mental health staff in those respective environments. Despite differences in content and organization these 2 RCs have the same overarching aim: to support recovery and coping by providing information and supporting self-management skills.

Both RCs have recently been evaluated by in-depth qualitative analyses. In Stockholm, course leaders, participants, and health care staff were interviewed individually with a focus on the perceived value of the course as well as promoters and barriers to implementation [22,23]. The value of Sagatun courses as perceived by participants was analyzed in focus group interviews, especially in relation to whether participants had an increased sense of independence and reduced dependence on public services, and whether the course had shown new ways of practicing user-provider collaboration [24]. In this study, we complement these findings with interviews with professional mental health staff in order to shed further light on how organizational factors, as well as their attitudes and practices, could further promote collaboration. The aim of this comparative study is to uncover whether there are differences between the embedded and freestanding RCs (1) in the perceived value of the RCs by participants, and (2) in psychiatry staff views on the benefits and challenges observed of the RCs and potential for collaboration.

## Methods

### Ethics Approval and Consent to Participate

Ethics approval was granted by the Regional Ethical Review Board of Stockholm (Dnr 2019‐03849 decision date October 22, 2019, with amendments Dnr 2020‐04604 decision date September 15, 2020, and DNr 2021‐04724 decision date September 20, 2021) and by the Norwegian Centre for Research Data (NSD nr 332381 decision date May 15, 2023). All procedures followed were in accordance with the Declaration of Helsinki of 1975, as revised in 2000. Written informed consent was obtained from all informants of this study.

### Design, Method Selection, and Analyses

The study design is an explanatory comparative case study. It uses the fact that the research group had access to 2 different user-led patient RCs, thus benefiting from increased variation in terms of contextual factors. The cases are from 2 countries, one being embedded in a mental health service and the other being a freestanding organization.

The case study research method is “an empirical inquiry that investigates a contemporary phenomenon within its real-life context; when the boundaries between phenomenon and context are not clearly evident; and in which multiple sources of evidence are used” [25]. The explanatory approach is used when the objective is to disclose causal links between real-life interventions and their outcomes, and experimental designs are not possible because of the complexity of events [25]. Those causal links will be demonstrated as empirical patterns, showing links between stakeholder actions, organizational and environmental conditions, and consequences observed.

### Theoretical Framework

This study compares how 2 RCs have been established, organized and run, and has the ambition to uncover benefits and challenges and seek explanations for those. That resembles the application of new knowledge or technologies and the introduction of new policies or practices. Greenhalgh et al [26] reviewed the literature on innovation and change in service organizations. They concluded that real and persistent change is achieved through an intricate interaction between the external environment and its stability, the properties of the innovation (change), the implementation strategy, and the actual change processes. Attention should be paid to the motives and actions of the stakeholders involved and their interrelationships, as well as the internal environment characterized by its structure, resources, leadership, and internal communication. To manage this complexity, a framework to guide data collection, analysis, and interpretation is warranted. Whipp and Pettigrew [27] proposed in their “strategic change management model” that 4 dimensions of change are studied and analyzed in relation to one another and over time: the local environment (context), the planned intervention (the content of the change), the implementation (the process of change), and outcomes, both in relation to predefined objectives and unintended consequences. This framework captures the contributing factors highlighted by Greenhalgh et al [26] but is simpler and thus a more practical tool for guiding data collection and analysis.

### Data Collection Methods

Individual and group interviews had been previously performed by the authors with RC participants as reported elsewhere [22-24]. Mental health staff were purposefully recruited to represent different services used by participants on both sites and interviewed individually or in groups (interview guides will be found in Multimedia Appendix 1). Documents consisting of advertisements, user information, course plans, and course material were studied. The data collection was guided by the Whipp and Pettigrew [27] framework in order to enable case descriptions with the following content:

1. Context: The mental health services for users in the cases, their professional staff and clinical practices, the relation between the service provider and the RCs, the funding of those, and policy and decision-making processes of relevance to the cases.
2. Content: The recovery-oriented activities described in detail.
3. Process: The ways in which those activities are performed, experienced by the users and their collaborating professionals, and the changes made in the clinical practices of the mental health services involved.
4. Outcomes: The results of the RCs, as described by the users and mental service staff, as well as their views on the perceived benefits and problems.

### Analysis

Interview data and documents were systematically examined by conventional content analysis [28]. Interviews were transcribed verbatim. The transcripts were subjected to conventional content analysis using an inductive approach. LA-A read the Stockholm transcripts and RJR read the Sagatun transcripts several times to reach immersion. They formulated independently meaning units with relevance to the study aims and covering all 4 dimensions of the theoretical framework. Those selected meaning units were then compared and discussed by LA-A and RJR and checked against the original transcripts. When consensus was reached the meaning units were labeled, grouped into categories (forming subthemes) and ultimately themes, separately for the 2 cases. Those were discussed by all authors, MB having detailed insights into the Stockholm case and HP into the Sagatun case. Consensus was reached on the appropriateness of the theme titles and related descriptions. As the views of the informants were used as an input to the characterization of the cases, dependent on other sources as well, member checking was not felt to be necessary.

Data from all sources, including the initial interviews with RC participants, were cross-checked and condensed into 2 thick case descriptions, structured according to the analytic framework [27]. Those are presented in full in Multimedia Appendices 2 and 3. Empirical patterns demonstrating the character and interrelations of the context, content, process, and outcome items were looked for and identified as tentative explanations to the benefits and challenges observed.

### Study Context: The Cases

#### Patient Recovery–Guided Activities at the North Stockholm County Department of Psychiatry

The department is responsible for psychiatric services in the northwestern part of Stockholm County, Sweden. It organizes in-hospital as well as outpatient specialty services. The department has been a front-runner in promoting user involvement and user-driven activities in mental health in Stockholm. It has employed full-time UICs since 2007; presently, there are 2 such UICs.

UICs have promoted patients’ rights and supported both in- and outpatients. Since 2016, service users (called “staff with user experience”) have been employed to provide peer support to patients.

UICs and SUEs established an RC offered to outpatients*,* organized in outpatient clinics. This “Patient School” consists of a series of 5 workshops given over 5 weeks covering the following themes: Psychiatry—how does it work?, Recovery—what is helpful?, Other resources in the society, Relations and disclosure, and Personal tools. The aim is to support users in self-managing their care and lives with a focus on social factors. Users are encouraged to actively exchange personal experiences. The course leaders invite, to each workshop, health care personnel from the psychiatry organization or researchers to act as coleaders and substance matter experts. To date, 12 of these courses have been offered with over 70 participants.

#### SR, Norway

SR in mental health and substance abuse is a private foundation, organizing recovery-inspired user education and social activities. Although users self-refer to the center, there is a close collaboration between the mental health service and the center. Sagatun functions as a regional resource base for the Inland region of Norway and a hub for recovery, working on local, regional, and national levels. Sagatun’s primary aim is to strengthen user involvement within the field of mental health and substance use, spreading information on a regional level and showing examples of good practice. The center is a low-threshold facility offering a meeting place and organizing social activities. On average, 50 persons visit the center regularly. “The Recovery Hub” is a collaborative project between the 6 regional user-led centers, including Sagatun, in Norway. These centers are meant to aid users’ and carer’s organizations, the educational sector, municipalities, and specialized mental health services in the adaptation of recovery principles. The Recovery Hub’s main objective is to provide persons with mental health and substance use issues and their next of kin with possibilities of recovery, self-efficacy, and the experience of a dignified life.

Sagatun offers an “SEC,” which resembles an RC. It comprises a course of 4 days; one 2-day workshop followed by a second workshop 14 days later. The themes are Empowerment, Communication, and User Involvement on an individual and systems level. Exercises are included that aim at strengthening self-confidence, spontaneous behavior, and the ability to set limits, as well as communicating those topics to others. Since the start in 2012, about 450 persons have attended the courses.

## Results

### Participant Views of the RCs

#### Stockholm “Patient School” (RC)

Sixteen participants of RC courses expressed in interviews that the following features of the RC were bringing value to themselves: the willingness of course leaders to share their own experiences, a sense of belonging and possibility to share with like-minded individuals, knowledge and practical skills acquired, and the opportunity to identify and experience new roles and behaviors. These experiences were empowering and enabled the transition from passive recipient of care to active partner, decreased feelings of stigma, and developed a sense that one’s identity is not defined by the mental health issue. These insights were seen to have the potential to lead to a more efficient use of available health care services [22].

#### SR (Self-Empowerment Course)

The study on participants’ views was a formal evaluation of the SEC that was launched in 2021 [24]. Six focus group interviews and 8 individual interviews were performed with former attendees during 2021 and 2022. Three overarching themes stood out as main findings of the study. Those were (1) From marginalization to independence: through increased knowledge and competence, the informants experienced becoming more self-sufficient and less dependent on the public services; (2) Knowledge of recovery: the informants saw a gap between their own needs and what the public services could offer, and that time was crucial to hold on to the recovery journey; and (3) Strengthening social networks: the course was acknowledged as an important arena for building bonds and relationships.

### The Views of Professional Staff on RCs

#### Stockholm “Patient School” (RC)

Eleven health and allied professionals working in Stockholm Psychiatry units as well as in municipal social psychiatry services were asked about their views on the RC and its possible impact on their practice. Both informants with and with no personal experience of the RC were approached.

Two psychologists and 1 social worker had managerial roles in psychiatry units. Five nurses and 1 occupational therapist had clinical positions in psychiatry units, 1 as a manager. One educationalist and 2 social workers were consultants in municipal social psychiatry units (Table 1).

**Table 1.** Stockholm informants.

Informant	Profession or education	Role in organization
0061	Psychologist	Manager, geropsychiatry unit
0067	Specialized nurse	Manager, psychiatry clinic
0069	Educationalist	Housing support consultant, social psychiatry
0070	Specialized nurse	Employment support consultant, social psychiatry
0071	Social worker	Employment support consultant, social psychiatry
1591	Psychologist	Deputy manager and clinician, psychiatry clinic
3337	Specialized nurse	Manager, emergency psychiatry unit
3715	Occupational therapist	OT, affective disorder clinic
4101	Specialized nurse	Psychiatry ward
9142	Nurse	Nurse, mobile emergency psychiatry unit
9983	Social worker	Manager, affective disorder clinic

Interviewees were aware of activities organized by SUEs, and a few had participated in RC sessions. Those who knew the RC well highlighted its benefits to patients and recommended their patients to participate. An in-depth analysis of the interviews identified 3 overarching themes: “Shifting away from a diagnosis-oriented healthcare system,” “Promoting co-care through education and peer-learning,” and “Creating a sustainable implementation of the RC within the psychiatry organization.” The first one relates to RC content, and the second and third themes relate to RC process. These staff views will be presented in detail in the section on the comparative analysis.

#### SR (Self-Empowerment Course)

This study examined what professionals working in different psychiatry units knew about the SEC. Individual interviews were conducted with 4 professionals working in either a Flexible Assertive Community Treatment team or a District Psychiatric Centre in April-June 2023. These informants were a nurse, special need nurse, or social worker. Three of the informants had higher education or a master’s degree in mental health, addiction, cognitive therapy, or violence risk assessment. Common for all the informants was that they were engaged in clinical work (Table 2).

**Table 2.** Sagatun informants.

Informant	Sex	Profession or education	Role and organization
A	Female	Nurse, master in mental health	Case manager in FACT^a^ team in a specialist health service
B	Female	Social worker, specialized in mental health	Case manager in FACT team in municipality
C	Female	Nurse, specialized in mental health and addiction	Nurse in District Psychiatry Centre
D	Male	Specialized SW^b^	SW in District Psychiatry Centre

a FACT: Flexible Assertive Community Treatment.

b SW: social worker.

These professional informants’ knowledge of the activities at SR and particularly the SEC and its perceived value was relatively scarce. Nevertheless, 3 of the informants had been on regular visits to the center and they had worked actively to introduce SR as a low-threshold service to patients they serve. None of the informants had inside information of the course, but all of them knew the existence of the course and knew of people having attended it. They all had the impression that this course represented a useful supplement to the existing public mental health services, both psychiatric specialist care and services provided locally by the municipalities. They were overall positive to this way of offering education and supposed that it gave the participants useful tools in order to manage both their illness and life in general. Transcripts of the interviews were scrutinized by thematic analysis. Two themes emerged—“Power balance” and “Knowledge gaps and overall challenges”—and those are presented comprehensively in the comparative analysis.

### Comparison of the 2 RCs

#### Overview

The differences and similarities between these 2 cases, as guided by the Whipp and Pettigrew [27] framework (context, content, process, and outcome), are exhibited in detail in Multimedia Appendix 4. The most important empirical findings will be presented in the following:

#### Context

In Stockholm, a *regional* self-governed public health authority runs specialized psychiatry services and primary care, which offers frontline psychiatry services by general practitioners and multidisciplinary mental health teams. *Municipalities* are responsible for social psychiatry, which offers professional support to people with long-standing mental health issues as well as sheltered housing and social benefits.

The RC is an *embedded* activity in the psychiatry organization. Persons with user experience are salaried employees who cooperate with professional staff. They have gradually established activities within the organization to support users. The activities are guided by recovery principles, but the ambition is to avoid creating a tension between educational activities and care provided by the psychiatry organization.

Sagatun works in an environment where a *state* health enterprise organizes psychiatry services on a *regional* basis, including outreach district psychiatry offered by multidisciplinary teams. *Municipalities* are in charge of primary care offering some therapeutic services and medication, as well as supported housing, home care, and vocational training. Social benefits are administered by a governmental agency.

Sagatun is a *freestanding*, user-led center with a *long history*, offering a *broad spectrum of activities* including a social meeting place. It is an open, *low-threshold service*, guided by recovery principles. In addition, it is a *regional resource and competence center* with the task to promote user involvement and influence. Its personnel include persons with user experience and some professional staff.

#### Content

The Stockholm course consists of 5 half-day workshops. Course leaders are persons with lived experience employed by the psychiatry organization. Invited experts contribute by providing information and giving advice. Practical information is provided on how the care system works and what practical support is available. Sharing of experience among participants is an important part of the course. Training in how to communicate one’s life situation is included. Activities cover a period of 5 years of experience and report 70 participants.

The Sagatun SEC consists of two 2-day workshops and additional 4-hour “recovery workshops” organized over a lengthy period of time. Course leaders are persons with lived experience who receive training and coaching by Sagatun staff. The focus is on recovery and communication, as well as exercises in coping strategies. Sagatun has over 10 years of experience and during that period, 450 persons have participated in the courses.

Mental health staff in Stockholm had concerns regarding the diagnosis-oriented health care system in Sweden, which mainly focuses on medication, sometimes to the extent of being the only treatment offered. This might harmfully strengthen a “patient identity” of service users potentially leading to an increased dependence on the health care system. They underscore the necessity for another approach in care that empowers users, encouraging them to become more active and committed to their recovery. To that end, the RC was seen as supporting a professional role that shifts away from this diagnosis-oriented approach (Interview theme: “Shifting away from a diagnosis-oriented healthcare system”). According to staff, meeting fellow users in different stages of recovery gave users new perspectives on one’s condition and help to reassess the identity as patient. This could break the vicious circle of dependence and promote recovery. RC as an educational activity would be a useful contrast to the care environment, showing the possibility to lead a fulfilling life despite challenges arising from their mental health condition. Users would also learn to navigate the complex psychiatric care environment.

The Norwegian mental health workers had a varying knowledge of SR and the SEC (“Knowledge gaps and overall challenges”). Despite this, everybody had positive views of SR. They described SR as a user-led meeting place and arena where users can meet peers, participate in activities in-house, establish friendships, and participate in excursions and a variety of courses. SR is seen as assisting people to be included in a community that helps them to take responsibility for their own lives. Some set up visits for their patients to SR and accompanied them there. SR is a meeting place offering users a sense of belonging, an opportunity to see other people in the same life situation and to be oneself. Informants tell about patients having benefitted greatly.

Some obstacles for service users on an individual level were observed, but challenges on a system level were also identified. The former were mental health status, social functioning, difficult economic situations, ongoing substance abuse, lack of motivation, and poor engagement. Serious mental illness and earlier failed treatment efforts diminished the interest to attend courses. There was a lack of information about SR and SEC among many service providers. The course program should be distributed to mental health units well in advance in order to make it possible for staff to provide information to potential course participants. Due to tight schedules, service providers might have limited opportunities to bring patients to SR. Follow-up of service users is increasingly organized by municipalities rather than mental health units, increasing the range of services that users need to be informed about. Both groups of mental health professionals had varying insights into RCs but were inspired to reflect on their value and their own working environments as to patient-centeredness and empowerment.

#### Process

Participants of the Stockholm course are recruited by spreading the word at wards and outpatient facilities, often by psychiatry staff. This RC has no dedicated resources, but salaried SUEs manage the courses. They lead the workshop discussions and share their own experiences frequently. Participants are encouraged to speak up, and some participants are more active than others.

SEC participants are directly recruited by Sagatun using advertisements and spreading information among users. The courses are part of Sagatun’s regular activities and funded through its budget. Persons with lived experience lead the courses which also include practical exercises. Special attention is given to verbal and nonverbal communication.

Stockholm staff interviewees reflected on how using an educational approach in care such as the RC can improve the collaboration between them as professionals and the users. They described the challenges encountered in psychiatric care, describing the roles and responsibilities they and users had. Staff were used to taking the lead in treating patients, particularly for those severely ill with limited understanding of their condition. This perceived professional responsibility can lead to an overly protective attitude, which could strengthen a traditional care model and hinder engaging users in service coproduction. The RC was seen as having the potential to align the expectations of users and professionals helping to express realistic goals. This mutual understanding could increase patient compliance to hospital care and treatment. Staff who understood their patients’ situation were more prone to recognize their potential and capabilities and thus engage in collaboration (“Promoting co-care through education and peer learning”). Improved internal cooperation between psychiatry units could be brought about by the RC, as staff participating would meet patients in different stages of their recovery and realize the input of their colleagues. While acknowledging the value of the RC, some interviewees mentioned that they adopted a similar educational approach. They encouraged patients to take an active role in their care during meetings between staff and users to design individual care plans.

To create conditions for a sustainable implementation of the RC within the psychiatry organization was seen by many staff as an important objective. They expressed a willingness to participate in the RC and support its activities. Better knowledge of the RC, especially concerning its benefits, among the professionals would help them to inform their patients and recruit participants. Increased awareness among staff would be necessary for a successful integration of the RC into the organization (“Creating a sustainable implementation of the RC within the psychiatry organisation”). That would ultimately be dependent on the political decision makers and psychiatry managers being willing to prioritize this activity and supply it with resources in a climate with a single-minded focus on service production. Convincing arguments showing the benefits of the RC would be crucial. To that end, systematic evaluations assessing outcomes, user satisfaction, and potential cost reductions are needed. Those would, although, be ill-suited to demonstrate positive impacts on users’ lives. Central to the sustainability of the RC is the employment of SUEs. The expertise and perspectives they bring to the organization are crucial.

Mental health staff interviewed about Sagatun reflected on the power balance between staff and users (“Power balance”). Little was said in relation to the SEC, but informants highlighted the importance of peer support workers (PSWs) in general and their role as “go-betweens” or “bridge builders” in mental health and substance abuse services. A majority also had personal experience of contacts with a PSW. They appreciated the knowledge and unique experience of PSWs. They communicated easily with patients, which made them useful in mental health services. In that way, they contributed to equalizing the power balance between service recipients and service providers as they use more of the patients’ perspective compared with the professionals. PSWs are increasingly employed in mental health services but do not have formal roles as defined by decision makers. This does not raise ethical concerns, but it is felt important that PSWs are part of the system and do not disregard psychiatric treatments.

Informants further specified that PSWs can contribute to treatment settings as they can more easily reach out to users and use their language and metaphors that are understandable to patients, thus contributing to normalizing situations and making those less threatening. This will make it easier to involve the user in designing treatment plans. Sometimes PSWs are closely related to a Flexible Assertive Community Treatment team, although in their own specific role. They are door openers to treatments and a social glue in treatment settings—but not therapists. As some patients tend to listen more to PSWs, everybody should be offered to meet a PSW.

In summary, the reflections of staff in both cases covered communication and interaction with service users far beyond RCs. Many focused on the importance of the organizational culture or care philosophy and facilitating and hindering conditions for fruitful coproduction, including contributing to an RC.

#### Outcomes

Valuable to Stockholm users were a sense of belonging and a possibility to share with like-minded. The knowledge and practical skills acquired were also valued. The opportunity to identify and experience new roles and behaviors was empowering and reduced the feeling of stigma. The improved knowledge of services and self-management skills was felt to lead to a more efficient use of available health care services.

Professional staff in Stockholm appreciated that information in the RC was delivered from a patient perspective. They thought that the course helps to normalize mental illness and reduce stigma. Those who participated in the courses were encouraged to take a more personal than professional role, which made communication easier. Staff interviewed thought that the RC could help to change the present diagnosis-focused care model to one based on service coproduction, which, supported by peer learning, would reduce user dependence and promote recovery and, at the same time, increase their compliance with medical treatment. To that end, they were willing to participate in an RC and contribute to raising awareness within the organization. Employees with user experience are the key to a successful integration of the RC but would, in addition, require that political decision makers and managers give high priority to that activity, including allocating resources.

Users participating in the Sagatun courses appreciated the social forum and its good atmosphere in the facility. Lasting contacts with peers and professional staff arose. Participants learned useful methods to set limits in life and acquired knowledge about their legal rights.

Mental health staff interviewed about Sagatun said that they had limited knowledge of its activities, particularly the SEC. However, some had made regular visits to the center, and they had worked actively to introduce SR. None had inside information about the course, but all interviewed knew the existence of the course and knew of people having attended it. Many mentioned how important the PSWs were and emphasized that they could bring forward the user’s perspective, communicate easily with users, and contribute to a more equal power balance between users and mental health professionals. A more active use of the SEC would require that service providers were better informed about Sagatun activities.

## Discussion

### Principal Findings

The principal findings in terms of similarities and differences as well as benefits and challenges when comparing an embedded versus a freestanding RC are the following:

The main similarities between recovery activities in Stockholm psychiatry and Sagatun are that both are led by persons with lived experience, focusing on supporting users in self-managing their lives through practical information and skills training. Sharing experience is an important part of the dynamics. Users experience that the courses are empowering and have the potential to reduce the stigma of mental illness. Mental health staff mostly appreciate the activities and inform their patients about the possibility to enroll, but they have limited knowledge about the content. The professionals feel that the activities potentially can lead to a more balanced relationship between professionals and users. However, no one mentions an impact on clinical practice or care arrangements.

There are major differences between the 2 sites. Stockholm psychiatry has almost a 20-year history of employing persons with lived experience with the task of enhancing user influence. They have introduced a number of routines to strengthen the position and rights of patients. One of the initiatives was the introduction of the RC.

Sagatun is a freestanding user-run facility established over 20 years and has a broad range of low-threshold services, including offering a physical meeting place and social activities in addition to self-empowerment support. It has a formal role as a regional resource and competence center and is, as such, well known both in mental health and local communities.

The Stockholm RC is an on-off experience; Sagatun offers continuity in terms of access to the center and participation in its various activities. Despite these differences, the perceived benefits are, by and large, the same. Users tell about benefits such as improved self-management skills. Mental health staff see the potential for a more balanced relationship between professionals and users, although there is little evidence of impact on professional roles and practices. In conclusion, these differences do not seem to be of importance in relation to perceived benefits, neither by users nor by mental health staff. Although staff appreciated the RCs and acknowledged the potential for improved collaboration with users, RCs did not seem to promote clinical practice changes.

When further scrutinizing outcomes, one has to look for nuances at best. Stockholm user participants emphasized as benefits the following: a sense of belonging, exchange of experiences, and practical knowledge helping to navigate the system (of care, social services, and support). They also felt a reduced social stigma. Sagatun clients appreciated the social forum offered by the center, acquiring knowledge of patients’ rights and long-lasting contacts with peers.

To summarize, we could not see any decisive differences between the embedded RC of Stockholm and the freestanding Sagatun facility. In both cases, the general knowledge among mental health staff about the activities was scarce. However, those who knew about the courses tended to recommend them to their patients. There was no evidence in staff interviews that the courses had influenced clinical practice. At best, RCs could have an impact on the care culture, reducing a diagnosis focus and promoting client-centeredness and coproduction, and be a supplement to formal mental health services.

However, the case comparison offers potential explanations to what could contribute to a successful RC, both offering benefits to users, improving communication and collaboration between users and mental health professionals, leading to practice changes and enhanced user-centeredness.

Having persons with user experience employed as staff in a mental health service would create a companionship that could promote a better understanding among professional staff and a more balanced relationship supporting recovery. It could also promote the internal spread of recovery-supporting activities of benefit to users, also showing how it can complement and potentially reduce the need for psychiatric care.

When organizations such as Sagatun offering recovery activities receive an institutional status in the mental health field, that will increase the general understanding of their benefits, have a potential for wider outreach, and lead to mental health services actively seeking cooperation and potentially integrating those into their service strategies.

Organizing courses that offer practical advice about the service system will increase users’ ability to navigate the system and self-manage. On the other hand, a well-thought-through psychoeducational approach with trained tutors or coaches offering skills training grounded in sensemaking and connectedness will create a firmer base for users to tackle challenges in everyday life. These are recommendations applicable to RCs in general, regardless of their organizational affiliation.

An initial assumption was that there would be differences in mental health staff views on the embedded and freestanding RC. In fact, there were few differences. Staff had, by and large, a positive image of the RCs but were not well informed about course activities. Those whose patients had participated in the courses had more knowledge and appreciated the contributions of the programs. Few saw the college as an activity that could complement their professional services, and there was no indication of impacts on their clinical practice. No one raised that RCs represent an educational effort which could complement clinical interventions. One reason might be that few of the staff had actively participated in an RC—this was the case in the embedded RC also.

In fact, most studies that have assessed the influence of RCs on mental health staff and their practice have been performed among professionals participating in RCs. Many of these studies focus on the value of coproduction during college activities. According to one of the studies, staff that had participated in RCs reported that the experience had changed their practices. They had gained increased understanding of service users’ relations, realities, and experiences, and modified their assumptions and biases concerning users. In addition, they had moved to “recovery-oriented practice acknowledging user self-determination, the importance of peer support, and promoting strength-based approaches” [29]. The same was reported in 2 studies performed among NHS mental health staff [30,31]. A recent systematic review found that coproduction in RCs affected professionals’ attitudes and the power dynamics between patients and practitioners and promoted a person-centered practice [32].

The value of working together was raised by Dalgarno and Oates [33]. As a result, senior clinicians had changed their perceptions of professional power and authority. A challenge, though, was the need to balance multiple roles and to see service users as colleagues. Reflecting on their clinical practice, these clinicians said that they had adapted their language to users and shared personal information with them. Another group of psychiatrists were aware of and had positive views of the RC concept. They had, however, concerns about its interaction with the medical model. There was a fear that some patients might reject their proposed medication. RCs were seen as complementary to mental health services [34].

Those observations were echoed by Crowther et al [35]. Developing new relationships with users led to valuing coproduction, changed perceptions of users, and increased job satisfaction. The authors also concluded that “a certain distance is needed in the relationship between the Recovery College and its host organisation if a genuine cultural alternative is to be created.” On the other hand, separate organizations might mean lower staff awareness about the RC and less potential impact on formal mental health services [34]. These are interesting propositions to compare with benefits and obstacles of NHS Trust affiliation (access to professional support and higher visibility vs lack of support due to competing priorities and limited autonomy due to bureaucratic rules) [16].

The Stockholm RC was “fully integrated” into the psychiatry organization and not only affiliated. The same challenges were identified, though: lack of management support and dedicated resources. Sagatun, on the other hand, is placed in the community and governed and run by persons with lived experience. It has been claimed that RCs working with local community mental health teams contributing with new ideas for activities could be a source of inspiration. RCs were able to draw on resources and the expertise of those teams [16]. Although Sagatun was known and appreciated by local mental health teams, these did not participate in RC activities. Crowther et al [35] found that a “third sector” RC expands its outreach in the community and has a positive influence on public attitudes to mental health. That could possibly be the case in the Norwegian setting but was not assessed in this study.

If closer collaboration with users, including participating in RCs, is a key to developing more user-centered practices in mental health organizations, how could mental health professionals' willingness to more actively collaborate with users and engage in RCs be promoted? One of the authors discussed how social psychology theories might guide such an effort [36]. Showing the benefits of user involvement to professionals and how those resonate with their professional beliefs and goals will have a positive effect on their attitudes toward coproduction. Engaging professional champions to lead coproduction will create social pressure and establish norms that will attract other professionals to participate.

### Methodological Considerations

This study has both strengths and weaknesses. Among strengths are that the authors have a decade of experience of the 2 sites with unlimited access and ongoing communication with RC staff. They also have detailed knowledge of the organizational context of the cases. An additional strength is the possibility to systematically compare RCs with differing organizational characteristics, situated in 2 countries: one is embedded in a psychiatry organization, which is part of a regional public health system, and the other is a freestanding charity governed by users with no formal contacts with public mental health services. A weakness is the limited size of the study in terms of informants. Being of qualitative nature, the results of this research have to be interpreted with caution and might have limited generalizability outside the studied regions.

### Conclusions

This multiple case study compared an RC embedded in a psychiatry organization with a freestanding, user-led RC. The RC contents showed only small differences; the embedded included more practical advice of formal services available, while the freestanding focused on the practice of social skills. The benefits experienced by participants matched those reported in the literature, including less experience of stigma, improved coping skills, and a reduction of service utilization.

Layperson SUEs employed in the psychiatry organization focused on safeguarding patient rights and improving patient involvement but were anxious to emphasize the value of evidence-based psychiatric care. Mental health staff had limited knowledge of the RCs on both sites. They still had interesting reflections on their workplaces in relation to recovery. In the psychiatry organization hosting the embedded RC, professional staff highlighted the “diagnosis-orientation” of their workplace and thought that a wider exposure to the RC could support a reorientation and promote a more patient-centered care culture and, as a result, improve collaboration between clinical units. PSWs (laypersons with user experience) engaged at the freestanding center were said by mental health professionals to be “go-betweens” and “bridge builders” to mental health services.

Overall, the differences between the RCs in views both by RC participants and mental health staff were small. The assumption at the outset of the study was that higher awareness and patient-professional collaboration would be found in the embedded RC but was not supported by these findings. Still, recent literature gives some advice on how to build on the insights from this study. A more recovery-oriented environment in mental health is promoted by engaged mental health professionals who have witnessed the benefits by actively participating in RCs. They realize the importance of collaboration with users and can act as champions to trigger change in clinical practice and care culture. The organizational form of the RC and its proximity to formal mental health services seem to be of less significance. People are more important than places.

## Supplementary material

10.2196/91076 Multimedia Appendix 1 Interview guides.

10.2196/91076 Multimedia Appendix 2 Region Stockholm Psychiatry Organisation Patient School, Stockholm, Sweden.

10.2196/91076 Multimedia Appendix 3 Sagatun Recovery, Hamar, Norway.

10.2196/91076 Multimedia Appendix 4 Comparative analysis.

## Abbreviations

NHS: National Health Service
PSW: peer support worker
RC: recovery college
SEC: self-empowerment course
SR: Sagatun Recovery
SUEs: staff with user experience
UIC: user-influence coordinator
