# Supplementary material for: Inside or Outside? A Qualitative Comparative Study of 2 Recovery Colleges in Sweden and Norway
Source: J Particip Med. 2026 Aug 11;18:e91076. doi: 10.2196/91076 (PMC13460067; doi:10.2196/91076)
Supplement: Supplementary file 1 [file jopm-v18-e91076-s001.docx]

**Multimedia Appendix 1**

**Interview guides**

Stockholm interview guide

Brief description of the study and registration of informed consent

Can you tell your profession/education and your present organisation and your role?

What is your view on collaboration between patients and professionals? Benefits and challenges?

Do you cooperate with User Influence Coordinators? What is their role? (Do you have an UIC as member of your management team?) – Benefits and challenges?

What do you know about the Patient School (PS)?

Can the PS have an impact on communication and collaboration with patients?

Do you think the PS is beneficial to patients?

Has the PS had on impact on patient care/your clinical practice? How?

Can your organisation support the PS? In what way?

What will it take to start a PS in your organisation?

What is the view of the professional staff on the PS (in general)?

Can you see any problems or challenges with the PS?

Is there something else you want to raise or mention?

Sagatun interview guide

(Interviewees were staff in specialist psychiatric services and interdisciplinary outreach teams=.

Brief information about the study and registration of informed consent.

Can you please tell me about yourself concerning the following;

Education

Place of work

Position and function

Primary work tasks

Years in professional work

Have you been working with peer support workers, and if so, what are your experiences?

What is your knowledge of Sagatun Recovery?

What are the primary aims of Sagatun Recovery?

Do you have any personal experiences with Sagatun Recovery – and in case, which?

How would you describe the impact of the work Sagatun Recovery does?

How does Sagatun Recovery impact your work and your organization?

Are you familiar with the Self-Empowerment Course at Sagatun Recovery?

What do you know about the purpose and aims of the course?

Have you had own experiences with the Self-Empowerment Course?

How do you consider the impact of the course?

Have the course had any impact on you and your organization?

How can this course have impact on the clinical work in your organization?

What is needed to increase attendance, and which barriers must be overcome?

In what way can your organisation become a supportive agent for Sagatun Recovery and the Self-Empowerment Course?

Is there something else you want to raise or mention?
