# Supplementary material for: Inside or Outside? A Qualitative Comparative Study of 2 Recovery Colleges in Sweden and Norway
Source: J Particip Med. 2026 Aug 11;18:e91076. doi: 10.2196/91076 (PMC13460067; doi:10.2196/91076)
Supplement: Supplementary file 2 [file jopm-v18-e91076-s002.docx]

**Multimedia Appendix 2**

**Region Stockholm Psychiatry Organisation Patient School, Stockholm, Sweden**

1. Context

The Stockholm Region (formerly known as the Stockholm County Council) is a self-governing public authority, elected by popular vote, that is responsible for providing primary, secondary, tertiary and community care for 2 million inhabitants in the Stockholm province. The province includes the Stockholm metropolitan area with suburbs showing big differences as to the socioeconomic status of their populations, several towns with big industries and an archipelago with sparsely populated local communities. Social services as well as care for the elderly and disabled, including housing services, rehabilitation and recreation, are the responsibility of the 16 municipalities (local public authorities) in the province. In terms of social protection, social welfare is also provided by the municipalities, whereas the Social Insurance Agency (granting, i.a., sick leave compensation and pensions) and the Employment Agency, assigning employments and paying unemployment benefits, are governmental agencies.

In 2004 all public primary and community care in Region Stockholm was consolidated into one organisation in 2004, the Stockholm Healthcare Services. It is presently responsible for services in primary care, psychiatry, geriatric care, outpatient care in some clinical specialities, advanced home healthcare with specialist support and rehabilitation.

Stockholm Psychiatry (SP) offers general psychiatric hospital and outpatient services for adults in five geographical areas. Eight hospital units have a total of 999 beds. The areas have established 180 psychiatric clinics for adults, children and adolescents. The inpatient and outpatient staff consists of psychiatrists, psychiatric nurses, psychologists and social workers, working individually and in multi-professional teams. Child and youth psychiatry is also organised as inpatient and outpatient units. Specialised clinics offer services for patients with substance abuse, eating disorders, affective disorders and newly diagnosed psychosis. SP is also responsible for forensic psychiatry in the region.

Primary care is organised in local health centres, offering mainly general practitioner services. Health centres also act as “first-line” psychiatric services. In addition to GPs, health centres employ clinical psychologists, psychiatric nurses and social workers. Health centres refer patients when needed to specialist services within SP. SP and primary care meet regularly in “open forums” to discuss collaboration.

Fifteen municipalities and 11 Stockholm city councils offer care and social support for persons with disabilities. Social psychiatry units, staffed by social workers, organise these services to disabled due to mental health or substance abuse issues. An important part of the service is sheltered housing and counselling. The right to residential care and social benefits is regulated by law and benefits are granted to clients by “social secretaries”, civil servants of the municipal social services administration. SP areas have formal agreements on collaboration with social psychiatry units in their catchment area. That collaboration is typically activated when a patient is discharged from a psychiatric hospital and is transferred into community care. In many instances, patients continue to visit their local psychiatry clinic after discharge.

Fifteen years ago SP units started to recruit persons with user experience to support to act as in-house consultants, participate in planning, to support patient involvement and promote the patient-centredness of services. Two categories of salaried co-workers were employed full-time or part-time, i.e. “user-involvement coordinators” (UICs, since 2007) and “staff with user-experience” (SUCs, since 2016). SUCs participate in organising activities for patients and act as mentors for patients on the psychiatric wards. Permanent positions for those were established in 2017.

2. Activities by staff with user experience

UICs have over the years taken a number of initiatives. All activities have been inspired by recovery theory. At the centre is the conviction that patients can recover from mental ill-health, and that it is the patient, active and in his or her own right, not a professional, who is best positioned to determine recovery. This will also empower the patient. Central to the theory is stigma. Negative symptoms, i.e. dysfunctions or impairments, are consequences of stigma connected to mental health disorders.

A weekly “Patient Forum” was introduced for in-patients in 2013. Those users are encouraged to raise issues and present ideas, which are conveyed to the unit and department managements, and lead frequently to quality improvement projects. In 2018 the UICs persuaded the North Stockholm department in 2018 to offer all users a private conversation, called “Daily Talks”, with a staff member every day. This was based on an initiative from the patient forum. The user choses the topic and the conversation can take up to 30 minutes. The lead UIC organised a systematic follow-up and performed a survey among users. Users felt that the conversations have had a positive impact on their care and empowered them. The professional staff was initially sceptical but learned to endorse this regular activity. Other activities have been a counselling group for UICs and SUEs, representation at the department’s Patient Safety Group and chairing the User Council with representatives of patient organisations and the department management. One UIC is a member of the department management team.

Recovery College: The “Patient School”

An educational activity, called the “Patient School” (PS) was established in 2018. It has been offered primarily to users registered at outpatient departments but is advertised also among in-patients.

The school is organised guided by the following principles: 1. The overarching aim is to promote recovery among participants. 2. The school is organised in facilities within Stockholm Psychiatry with the support of its leadership. 3. Course leaders are UICs and SUCs employed at SP. 4. Participants are encouraged to share of personal experience to be left to others to reflect upon, but no recommendations (e.g. in terms of coping strategies) are made. and reflect on those narratives but referring to evidence-based psychiatric practices (“this has worked for X, but you should critically assess whether will suit you”).

The aim of the Patient School is to support users’ in self-managing their care and lives with a focus on social factors. Users form a group that meets to five workshops. Knowledge and practical information are presented and discussed for each of the following themes: Psychiatry – how does it work?; Recovery – what is helpful?; Other resources in the society; Relations and disclosure; and Personal tools. The course leaders invite, to each workshop, health care personnel from Stockholm Psychiatry or researchers to act as co-leaders and substance matter experts. Written tutorial material was provided for all workshops. Since the start 12 courses have been organised with over 70 participants.

The School was open to all users that learn about the activity. UICs and SUEs actively spread information among users. Written material was available at outpatient departments and wards. Professional staff did, when informed and interested, tell their patients about the School. However, if clinical staff ha no information or lacked contacts with UICs and SUEs, they were not able to support recruitment efforts. Negative attitudes of staff on the School were observed in some instances; they were suspicious of employees with user experience and questioned the value of the PS or had doubts about the value of patient involvement and empowerment. These were also obstacles to the recruitment of participants.

Stockholm Psychiatry funded the School by allowing salaried UICs and SUEs to act as course leaders, providing venues and educational material and some funding for external experts. However, there was no dedicated budget for the School. Clinical staff had difficulties to contribute as their participation was in conflict with service providing targets. The Region’s funding model reimburses units per clinical services performed.

The course leaders moderated the workshops, distributed and explained the course material and supported external experts. They actively shared their own lived experience, which encouraged participants to join the discussions and tell their own. The course leaders gave everybody space and paid attention to participant feedback. Organisers, participants and clinical staff summarised their views on the preconditions for successful implementation as follows: active recruitment of participants at wards and outpatient departments, information freely available in the same locations, a dedicated budget and venue for course activities, active moderation of discussions during courses, responding to participant needs, adjusting the group dynamics, and paying attention to the feedback by course participants.

3. Perceived value

*Participants* found the following features of the Patient School as bringing value to themselves: the willingness of course leaders to share their own experiences, a sense of belonging and possibility to share with like-minded, knowledge and practical skills acquired, and the opportunity to identify and experience new roles and behaviours. These experiences were empowering and enabled the transition from passive recipient of care to active partner, decreased feeling of stigma and developed a sense that one’s identity is not defined by the mental health issue. These insights were seen to have the potential to lead to a more efficient use of available healthcare services.

When discussing if the Patient School did improve the knowledge and skills of participants *clinical staff* that had been present at sessions described that all information was delivered from a patient perspective, something that they as professionals were not able to do. They also felt that the objective of the PS to normalise mental illness and reduce stigma was fulfilled. Course leaders emphasised that their intention was to create a safe space also for participating healthcare professionals enabling them to take a more personal role, compared to their usual professional role, when they visited the Patient School. However, staff said they had the same role in the Patient School as when they met patients in the care setting.

4. Clinical staff views of the Patient School in relation to their professional role and clinical practice

Health professionals working in Stockholm Psychiatry units as well as in municipal social psychiatry services were asked about their views on the Patient School and its possible impact on their practice. Both informants with and without personal experience of the PS were approached.

Thematic analysis of these interviews provided three overarching themes and a number of sub-themes.

The theme “Shifting away from a diagnosis-oriented healthcare system” sheds light on interviewees' concerns regarding the diagnosis-oriented healthcare system in Sweden, which mainly focus on medication, sometimes to the extent of being the only treatment offered. This might harmfully strengthen a “patient identity” of service users potentially leading to an increased dependence on the healthcare system. They underscore the necessity for another approach in care that empowers users, encouraging them to become more active and committed to their recovery. The PS was described as serving a role that shifts away from this diagnosis-oriented approach. Meeting users in different stages of recovery will give new perspectives on one’s condition and help to reassess the identity as patient. This will break the vicious circle of dependence and promote recovery. PS as an educational activity would be a useful contrast to the care environment, show the possibility to lead a fulfilling life despite challenges despite their diagnosis. Users would also learn to navigate the complex psychiatric care

“Promoting co-care through education and peer-learning” was a theme that highlighted the interviewees’ reflections on how using an educational approach in care such as the PS can improve the collaboration between them and users. They described the challenges encountered in psychiatric care, describing the roles and responsibilities they and users have. Staff are used to taking the lead in treating patients, particularly for those severely ill with limited understanding of their condition. This perceived professional responsibility can lead to an overly protective attitude. This will strengthen a traditional care model and hinder engaging users in service co-production. The PS was seen as having the potential to align the expectations of users and professionals helping to express realistic goals. This mutual understanding would increase patient compliance to hospital care and treatments. Staff who understand their patients’ situation is more prone to recognise their potential and capabilities and thus engage in collaboration. Improved internal cooperation between psychiatry units could be brought about by the PS, as staff participating would meet patients in different stages of their recovery and realise the input of their colleagues. - While acknowledging the value of the PS some interviewees mentioned that they adopted a similar educational approach. They encourage patients to take an active role in their care during meetings between staff and users to design individual care plans.

“Creating a sustainable implementation of the PS within the psychiatry organisation” was seen by interviewees as an important objective. They expressed a willingness to participate in the PS and support its activities. Better knowledge of the PS, especially concerning benefits, among the professionals would help them to inform their patients and recruit participants. Increased awareness among staff would be necessary for a successful integration of the PS into the organisation. That would ultimately be dependent on the political decision-makers and psychiatry managers being willing to prioritise this activity and supply it with resources in a climate with single-minded focus on service production. Convincing arguments showing the benefits of the PS would be crucial. To that end, systematic evaluations assessing outcomes, user satisfaction and potential cost reductions are needed. Those would, though, be ill-suited to demonstrate positive impacts on users’ life. Central to the sustainability of the PS is the employment of staff with user experience. The expertise and perspectives they bring to the organisation is crucial.

Finally, some interviewees emphasised that the PS is more suitable for patients like newly diagnosed who would learn at an early stage to navigate the system, and long-stay in-patients who need support to recover and adjust to independent living. Others believed that the PS could be valuable for all patients, regardless of their stage in the recovery journey. Good collaboration with staff is needed to avoid harmful impact on group dynamics, which might make it less suitable to invite very ill patients.
