# Supplementary material for: Inside or Outside? A Qualitative Comparative Study of 2 Recovery Colleges in Sweden and Norway
Source: J Particip Med. 2026 Aug 11;18:e91076. doi: 10.2196/91076 (PMC13460067; doi:10.2196/91076)
Supplement: Supplementary file 3 [file jopm-v18-e91076-s003.docx]

**Multimedia Appendix 3**

**Sagatun Recovery, Hamar, Norway**

1. Context

Mental health services in the Inlandet province are organised by the government-owned provincial health enterprise (Sykehuset Innlandet Helseforetak) in collaboration with the municipalities. The health enterprise provides specialised inpatient and outpatient psychiatric services. Specialised inpatient treatment is mostly provided by the psychiatric hospitals, which traditionally has been situated in rural areas, with the underlying philosophy to provide green environments for the patients while in treatment. The tendency in recent establishments of new psychiatric hospitals is to co-locate these services with big somatic hospitals. In addition, during the last 20 years we see that most psychiatric hospitals have developed district psychiatric centres (DPS) offering both in- and outpatient treatment. These centres functions as “a bridge” between the psychiatric hospitals and the municipalities.

Outreach services in mental health and addiction care have been developed during the last 15 years, and are provided by multidisciplinary teams of psychologists, social workers, psychiatric nurses, assistant nurses as well as occupational therapists. During the last 8-10 years peer support workers have also been employed alongside the professionals. These teams often apply the ACT-model (Assertive Community Treatment) or more recently the FACT model (Flexible Assertive Community Treatment). Almost 100 such teams have been established nationwide during the past 10-12 years. This development is a consequence of governmental policy to reduce in-patient treatment and put more responsibility to the municipalities and now play an important role in Norwegian psychiatry and mental health care. These services are organised in collaboration between municipalities and psychiatric specialist care (part of the health enterprise).

Primary care is provided by privately practising general practitioners (GP), funded by the government (to the biggest part) and the municipalities, providing medical and social services to mental health patients. GPs can offer therapeutic conversations, treatment, medication and follow-up. GPs can also inform about other relevant services in the municipality, such as low-threshold centres, follow-up by mental health teams, short-term treatment by psychologists, supported housing, home care and vocational training. Some of these services need a referral from a GP, but mostly these services are available without referral. Also, most municipalities are organized to offer services to those having co-occurring problems (both mental health and substance use), as mental health and substance use often are linked to each other. Furthermore, the municipalities also have the responsibility for care for the elderly and disabled, including sheltered housing and home healthcare. Social security and benefits are administered by the state Work and Welfare Authority (Norwegian abbreviation: NAV).

2. Sagatun Recovey

Sagatun Recovery (a user-led low threshold centre) in Hamar is a free-standing facility organising a range of recovery supporting activities, including a recovery college. It is one of six regional user-led centres in Norway, established to promote patient involvement and influence in mental health services as well as advice on user-led activities in their regions. Sagatun Recovery is owned and managed by a foundation, established by the Association for Mental Health in Hedmark, a regional NGO. According to its by-laws users and user organisation representatives occupy a majority of seats on the board of the foundation. The foundation receives donations and subsidies from the state and municipalities and takes the responsibility for funding the centre and its activities. The centre organises regional services, but it is open also to people outside the Innlandet region. It is meant to be a social meeting place offering people the opportunity to grow and develop on their own conditions. The centre offers recovery-supporting activities and work training and is a resource and competence centre for user-participation, user-direction and empowerment. It is a meeting place for users and mental health and social care professionals. Activities are recreation, social contacts, work training, and courses that support competence development and self-empowerment. The main target group is people with mental health and substance abuse challenges and their next-of-kin, but anyone is welcome.

The central basic principle guiding the centres activities is that everybody has important resources, can contribute and that all participate based on their own preconditions and potential. No-one asks about diagnoses and users themselves define what services they want to utilise and what activities to engage in.

The core idea of the centre is to support empowerment and recovery among its users. This is best done by embracing the power that lies in one’s own experience and knowledge about what can contribute to an improvement of a challenging life-situation. The centre offers activities, participation, competence development that will help individuals to find their interests and resources, experience meaning and coping, regain their belief in themselves and support their capacity to manage their lives. The centre staff consist mainly of persons with user-experience and next-of-kin, of professionals representing mental health, substance abuse and music therapy and administrative staff, fourteen in all. A User Board is elected by those participating in centre activities. It takes part in the planning and development of the centre, informs about the centre and organises recreational activities. The Board chair is a member of the Foundation Board.

The centre is situated in a villa in Hamar, called the “House of Dreams”. It is open during the day with some activities in the evenings. The centre runs a cafeteria, organises activities and runs projects with external funding. Various activities aim at creating joy and supporting coping. Most of these are organised by users and volunteers forming a supportive work community. The Work and Welfare Authority has licensed Sagatun Recovery for work training and activities required by unemployment benefits receivers. Outdoor activities like hikes and camps are numerous. A music workshop and studio offer the opportunity to users to form bands, write music, rehearse and perform. A creative workshop focuses on art and handicraft. In the “sponsor project” sponsors (mentors) are educated to support peers and help them to feel part of the community and safe as they strive to cope with exclusion and loneliness.

As a regional centre Sagatun Recovery is a “flagship for recovery” providing advice and support to organisations working with users. It provides them with knowledge and skills to enhance user participation, recovery and empowerment. The objective is to support users and next-of-kin to increase their capacity to manage their life-situation and influence public services and the society. The centre participates in research and development, especially in participatory research in collaboration with several universities in Norway.

Recovery College: “Self-Empowerment Course”

Sagatun Recovery organises, under an umbrella called “Toolbox for user participation”, several “Self-empowerment courses” annually, an activity that started in 2012, and has over the years been well established. In resembles in all dimensions a Recovery College. In 2021 the centre offered 9 courses to a total of 55 participants.

The course comprises of four days; one two-day workshop followed by a second workshop 14 days later. The themes presented and discussed are User participation, Communication, and Recovery and Empowerment on an individual and systems level. Exercises are included that aim at strengthening self-confidence, spontaneous behaviour and ability to set limits, as well as communicating those topics to others. Since the start in 2012 about 450 persons have attended the courses. Regular Recovery workshops focusing on different themes are organised. One theme is “Introduction to recovery” which introduces the CHIME framework for personal recovery (Connectedness, Hope and optimism, Identity, Meaning, Empowerment). Other themes are Hope, Connectedness and Meaning and Physical activity, Music and Creativity, and how those emotions and activities influence recovery. These workshops last four hours with introductions on theory and experience, walk and talk in pairs and group reflections.

Course leaders are persons with user-experience within the Sagatun Recovery staff. They are recruited through the Self-Empowerment Course knowledge form the use of Sagatun Recovery, or through our cooperation partners in the regional user centres in Norway. They are being trained in special designed process management courses and process leader gatherings. Furthermore, they receive guidance and being evaluated by experienced course leaders. A typical course starts with daily with a sort of “weather forecast” to be able to scan the state of mind of the participants. Discussions are facilitated through physical exercises as a training activity to ease the ability to make choices. Leaders sometimes share their experiences by being initiators of the conversations. The following skills are trained; User involvement on an individual level, communication, recovery and empowerment. They also undergo training in patient rights concerning their own feedback on treatment and what is necessary to develop good communication with different parts of the services. It is also stressed the importance of how to start up their own recovery process, by finding strength and power within themselves. Issues of communication can be caused by various factors. Several disapproves body language as an essential part of all human communication. This also receives special attention during the courses. The course stresses that “You have to make your own borders visible if you expect that others shall be aware of them”.

All courses have been evaluated using a standard form to be filled in by participants. They generally are very satisfied with the course and describe it as “a good learning experience”. A good balance between theory and practice has usually been achieved. Participants appreciate the opportunity for communication between users and centre staff. The information conveyed about user participation was useful and discussions in the reflection groups were fruitful.

3. Perceived value of the Self-Empowerment Course

A formal evaluation of the course was launched in 2021. Six focus group interviews and eight individual interviews were performed with former attendees during 2021 and 2022. The findings were mainly in line with earlier participant feedback (through evaluation forms). The course as a social forum was especially appreciated, participants mentioned its “good atmosphere” and that lasting contacts were created, including those with professional staff. Participants learned useful methods to set limits and learned about their legal rights, one example being the right to have an individual work plan. Three overall themes stood out as main findings in the study; 1) Independence and autonomy, 2) Different ways to practice user involvement and 3) Concrete suggestions to improve the courses. 4.

4. Clinical staff views of Sagatun Recovery and the Self-Empowerment Course [in relation to their professional role and clinical practice?]

To which extent professionals working in different psychiatry units knew about the Self-Empowerment course and the significance of these courses was explored conducting individual interviews with four professionals working in either a FACT-team or a DPS (District Psychiatric Centre) in April-June 2023. These informants a nurse, special need nurse or social worker. Three of the informants had higher education or a master‘s degree in mental health, addiction, cognitive therapy or violence risk assessment. Common for all the informants was that they were engaged in clinical work.

To summarise, the informants’ knowledge of the activities at Sagatun Recovery and particularly the Self-Empowerment Course and its perceived value were relatively scarce. Nevertheless, three of the informants had been on regular visits to the centre and they had worked actively to introduce Sagatun Recovery as a low-threshold service to patients they serve. None of the informants in had inside information of the course, but all of them knew the existence of the course and knew of people having attended it. They all had the impression that this course represents a useful supplement to the existing public mental health services, both psychiatric specialist care and services provided locally by the municipalities. They were overall positive to this way of doing education and supposed that it gave the participants useful tools in order to manage both their illness and life in general.

Transcripts of the interviews were scrutinised by thematic analysis. Two themes emerged - power balance, and knowledge gaps and overall challenges – and those are presented below.

Power balance

Informants raised the importance peer support workers (PSW) and their role as “go-betweens” in mental health and substance abuse services. A majority had also personal experience of contacts with SW. They appreciated the knowledge and unique experience of PSWs. They communicate easily with patients which makes them useful in mental health services. In that way they contribute to equalising the power balance between service recipients and service providers as they employ more of the patients’ perspective compared to the professionals. PSWs are increasingly employed at mental health services, but do not have formal roles as defined by decision-makers. This does not raise ethical concerns, but its is felt important that PSWs are part of the system and do not disregard psychiatric treatments.

Informants further specify that PSWs can contribute to treatment settings as they can more easily reach out to users, use their language and metaphors understandable to patients, and, thus contribute to normalising situations and making them less threatening. This will make it easier to involve the user in designing treatment plans. Sometimes PSWs closely related to a FACT team, although in their own specific role. They are door-openers to treatments a social glue in treatment settings – but not therapists. As some patients tends to listen more to PSWs everybody should be offered to meet a PSW.

Knowledge gaps and overall challenges

The informants had varying knowledge Sagatun Recovery (SR) and the Self-empoverment course (SEC). Despite this, everybody had positive views of SR. They describe SR as a user-led meeting place and arena where users can meet peers, participate in activities in-house, establish friendships, and participate in excursions and a variety of courses. SR is seen as assisting people to being included in a community which helps them to take responsibility for their own lives. Some set up visits for their patients to SR and accompany them there. SR is a meeting place offering users a sense of belonging, to see other people in the same life situation and to be one-self. Informants tell about patients having benefitted greatly.

Some obstacles for service users on an individual level were observed, but challenges on a system level were also identified. The former were mental health status, social functioning, difficult economic situation, ongoing substance abuse, lack of motivation and poor engagement. Serious mental illness and earlier failed treatment efforts diminish the interest to attain courses. On the systems level there is a lack of information about SR and the Self-empowerment course among many service providers. The course programme should be distributed to mental health units well in advance in order to make it possible to provide information to potential course participants. Due to tight schedules service providers might have limited opportunities to bring patients to SR. Follow-up of service users is increasingly organised by municipalities rather than mental heal units, increasing the range of services to be informed.
