# Supplementary material for: Inside or Outside? A Qualitative Comparative Study of 2 Recovery Colleges in Sweden and Norway
Source: J Particip Med. 2026 Aug 11;18:e91076. doi: 10.2196/91076 (PMC13460067; doi:10.2196/91076)
Supplement: Supplementary file 4 [file jopm-v18-e91076-s004.docx]

**Multimedia Appendix 4**

**Comparative analysis**

Similarities and differences

|  | Stockholm | Sagatun |
| --- | --- | --- |
| Outer context | Regional public health authority  Self-governing  Regional tax funding  Provides primary, secondary and tertiary care including psychiatry (adult, child and adolescent) in-patient and outpatient services  Specialised clinics for substance abuse and some diagnoses  Primary care organised in local health centres also offering “first-line psychiatric services” (Salaried GPs, psychologists, psych nurses, social workers).  Municipalities responsible for “social psychiatry” – social support, social benefits, sheltered housing and counselling for persons with long-term service needs. Benefits are granted in an administrative process (municipality)  Summary:  Regional self-governed public health authority specialised psychiatry services and primary care (front-line psychiatry services by GPs and multidisciplinary mental health teams.  Municipalities responsible for “social psychiatry” including sheltered housing and social benefits. | State-run and financed “health enterprise” providing secondary care including psychiatry (co-located with somatic hospitals) – in-patient and outpatient services  Organises outreach “District psychiatric services” offering in- and out-patient services by FACT teams (psychologists, social workers, psych nurses, assistant nurses, OTs).  Municipalities organise primary care (private practitioner GPs who offer therapeutic conversations, medication and follow-up) and low threshold centres, follow-up by mental health teams, supported housing, home care and vocational training.  Social security and benefits are administered by State work and welfare authority (NAV).  Summary:  State health enterprise organising psychiatry services, including outreach district psychiatric services with multidisciplinary FACT teams.  Municipalities in charge of primary care offering some therapeutic services and medication, and supported housing, home care and vocational training.  Social benefits administered by NAV (state). |
| Inner context | Stockholm psychiatry employs persons with user experience since 2010 (2017) (user-involvement coordinators, UICs, and staff with user experience, SUE)  UICs/SUEs organise weekly Patient Forums in the hospitals, patient-prof staff “Daily Talks”, lead User council, perform regular surveys among users, liaise with SP clinical managers.  SP recovery activities are offered to SP service users.  Philosophy:  “At the centre is the conviction that patients can recover from mental ill-health, and that it is the patient, active and in his or her own right, not a professional, who is best positioned to determine recovery. This will also empower the patient. Central to the theory is stigma. Negative symptoms, i.e. dysfunctions or impairments, are consequences of stigma connected to mental health disorders.  Summary:  Embedded activity in the psychiatry organisation. Persons with user-experience are salaried employees who gradually has established activities within the organisation to support users. Guided by recovery principles, but ambition to avoid creating a tension between offered activities and care provided by the psychiatry organisation.  Cooperate with professional staff. | Sagatun is a free-standing organisation resembling a charity, owned and managed by a foundation, established by an NGO. The majority of the board represent users. It works in a facility called “The House of Dreams”.  The foundation is funded by subsidies from the state and municipalities as well as donations.  Sagatun is one of six regional user-led centres with the task of promoting patient involvement and influence in mental health. It is a resource and competence centre for user-participation, user-direction and empowerment. It is freely open for everybody and is a low-threshold service, not requiring a referral and a no medical record is kept.  The centre staff consists of persons with user-experience (PUE)and next-of-kin, mental health, substance abuse and music therapy professionals and administrative staff, fourteen in all.  PUEs have a central role in organising centre activities, meet patients, and act as a bridge to psychiatry services. They are seen as “part of the system”, but with their distinct role (they are not therapists!) .  The centre offers recovery-supporting activities to users in the Innlandet region (is open to other participants also) and gives advice on user-led activities to regional actors. The centre is a social meeting place (incl. cafeteria) for users, it offers recovery-supporting activities (outdoor activities, music workshop and studio, creative workshop focusing on art and handicraft) and a smaller degree of work training licensed through NAV  Philosophy:  “The central basic principle guiding the centres activities is that everybody has important resources, can contribute and that all participate based on their own preconditions and potential. No-one asks about diagnoses and users themselves define what services they want to utilise and what activities to engage in.  The core idea of the centre is to support empowerment and recovery among its users. This is best done by embracing the power that lies in one’s own experience and knowledge about what can contribute to an improvement of a challenging life-situation. The centre offers activities, participation, competence development that helps individuals to find their interests and resources, experience meaning and coping, regain their belief in themselves and support their capacity to manage their lives.  Summary:  Free-standing, user led centre with a long history, broad spectrum of activities including social meeting place, being an open, low-threshold service, guided by recovery principles, in addition, regional resource and competence centre to promote user-involvement and influence, personnel include persons with user-experience and professional staff. |
| Content | *The Patient School (PS)*  is organised guided by the following *principles*:  1. The overarching aim is to promote recovery among participants.  2. The school is organised in facilities within Stockholm Psychiatry with the support of its leadership.  3. Course leaders are UICs and SUCs employed at SP.  4. Participants are encouraged to share personal experience but course leaders do not generalise those narratives or give recommendations (e.g. in terms of coping strategies)  The *aim* of the Patient School is to support users’ in self-managing their care and lives with a focus on social factors.  *Course leadership*:  Course leaders are UICs and SUCs employed at SP  *Course content*:  Five half-day workshops during which knowledge and practical information are presented and discussed for each of the following themes: Psychiatry – how does it work?; Recovery – what is helpful?; Other resources in the society; Relations and disclosure; and Personal tools. The course leaders invite, to each workshop, health care personnel from Stockholm Psychiatry or researchers to act as co-leaders and substance matter experts.  Written tutorial material was provided for all workshops.  Since the start in 2018 *12 courses have been organised with over 70 participants*.  Summary:  Five half-day workshops. Course leaders with lived experience and invited experts. Practical information provided on how the care system works and what practical support is available. Sharing of experience and training in how to communicate one’s life situation. Five years of experience and 70 participants. | *Self-empowerment Course (SC)*  Under an umbrella called “Toolbox for user participation”, “Self-empowerment courses” are offered.  *Course leadership*:  Course leaders are persons with user-experience within the Sagatun Recovery staff. They are being trained in specially designed process management courses and process leader gatherings. They receive guidance and are evaluated by experienced course leaders.  *Course content*:  The course comprises of four days; one two-day workshop followed by a second workshop 14 days later.  The following skills are trained: User involvement on an individual level, communication, recovery and empowerment. Training in patient rights is included.  Exercises are included that aim at strengthening self-confidence, spontaneous behaviour and ability to set limits, as well as communicating those topics to others. Advice is given on how to start up their own recovery process, by finding strength and power within themselves  Regular four-hour Recovery workshops focus on different themes like Hope, Connectedness and Meaning; and Physical activity, Music and Creativity, and how those emotions and activities influence recovery. Introduction to recovery introduces the CHIME framework for personal recovery (Connectedness, Hope and optimism, Identity, Meaning, Empowerment)..  Since the start in 2012 about *450 persons have attended the courses*.  Summary:  Two two-day workshops and additional four-hour “recovery workshops”.  Course leaders with lived experience who receive training and coaching.  Focus on recovery and communication, exercises in coping strategies. Over ten years of experience and 450 participants. |
| Process | Information and recruitment of participants:  UICs and SUEs actively spread information among users. Written material is available at outpatient departments and wards. Professional staff do, when informed and interested, tell their patients about the School. Negative attitudes of staff on the School were observed in some instances; they were suspicious of employees with user experience and questioned the value of the PS or had doubts about the value of patient involvement and empowerment. These were also obstacles to the recruitment of participants.  Resources:  Stockholm Psychiatry funds the School by allowing salaried UICs and SUEs to act as course leaders, providing venues and educational material and some funding for external experts.  However, there is no dedicated budget for the School. Clinical staff has difficulties to contribute as their participation is in conflict with service providing targets.  How the course is delivered:  The course leaders moderated the workshops, distributed and explained the course material and supported external experts. They actively shared their own lived experience, which encouraged participants to join the discussions and tell their own. The course leaders gave everybody space and paid attention to participant feedback.  Organisers, participants and clinical staff summarised their views on *the preconditions for successful implementation* as follows: active recruitment of participants at wards and outpatient departments, information freely available in the same locations, a dedicated budget and venue for course activities, active moderation of discussions during courses, responding to participant needs, adjusting the group dynamics, and paying attention to the feedback by course participants.  Summary:  Participants are recruited by spreading the word at wards and outpatient facilities, often by staff.  The PS has no dedicated resources, but salaried UICs/SUEs manage the courses.  UIC/SUEs lead the workshop discussions and share their own experiences frequently. Participants are encouraged to speak up, with a risk of a few dominating. | Information and recruitment of participants:  Sagatun recovery (SR) recruites participants to their Self-empowerment Course (SC) by advertising locally. This implies spreading information both internally at SR and in district psychiatric centers (DPS).  SR works also closely with other user-led and low-threshold centers, and often takes initiatives to arrange courses for such centers. Participants sign up or show interest in attending the course on their own initiative.  Resources: The Self-empowerment Course is, like other Sagatun activities, funded from the centre’s overall budget.  How the course is delivered:  Firstly, the courses focus on psychoeducation concerning communication, rights and responsibilities. Secondly, the course focus on experience-based activities. This entails activities the participants work on individually, two-and-two and in small groups.  The activities are designed so that the participants can be trained in issues concerning relationships and communication. This involves training in being able to express their meanings, to convey them to others and receive feedback and being able to make choices for themselves.  Summary:  Participants are directly recruited by Sagatun recovery using ads and spreading information.  The Self-empowerment courses are part of Sagatun’s regular activities and funded through its budget.  PUEs lead the course which also includes practical exercises. Special attention is given to verbal and non-verbal communication. |
| Outcomes | Perceived value:  Users  Valuable were: the willingness of course leaders to *share their own experiences*, a *sense of belonging and possibility to share with like-minded*, *knowledge and practical skills* acquired, and the *opportunity to identify and experience new roles and behaviours*.  These experiences were *empowering* and enabled the transition from passive recipient of care to active partner, *decreased feeling of stigma* and developed a sense that one’s identity is not defined by the mental health issue. These insights were seen to have the potential to lead to a *more efficient use of available healthcare services*.  Clinical staff views:  When discussing if the Patient School did improve the knowledge and skills of participants clinical staff that had been present at sessions described that all information was delivered from a patient perspective, something that they as professionals were not able to do. They also felt that the objective of the PS to normalise mental illness and reduce stigma was fulfilled. Course leaders emphasised that their intention was to create a safe space also for participating healthcare professionals *enabling them to take a more personal role*, compared to their usual professional role, when they visited the Patient School. However, staff said *they had the same role in the Patient School as when they met patients in the care setting*.  Interviewees aired concerns that *Swedish psychiatry is diagnosis-oriented with a heavy focus on medication*. That can contribute, over time, to *an increased dependency of patients on the care system*. They underscore the *necessity for another approach* in care that *empowers users*, encourages them to become more active and committed to their recovery. The PS was described as serving a role that *shifts away from this diagnosis-oriented approach*.  The health professionals recognised that *an educational approach in care such as the PS can improve the collaboration between them and users*. This could *change the roles and responsibilities of professionals and users and promote user-centric care*. They identified as another positive effect *improved collaboration between psychiatry units*. The *important role of staff with user experience in promoting this collaboration*.  Clinical staff interviewed expressed their *willingness to participate in the PS* and support its activities but stressed the importance of receiving sufficient information about the activities. They emphasised that *the success of a sustained PS implementation depends on policymakers and their commitment, as well as the contribution of staff members with user experience*. In order to influence decisions made by politicians, it would be important to be able to present evaluations demonstrating the effectiveness of the PS through measurable outcomes.  Summary:  *Valuable to users* were:  Sense of belonging and possibility to share with like-minded, knowledge and practical skills acquired, and the opportunity to identify and experience new roles and behaviours – all were empowering and reduced stigma.  Improved knowledge and skills lead to a more efficient use of available healthcare services.  *Professional staff observations*:  All PS information was delivered from a patient perspective.  PS helps to normalise mental illness and reduce stigma. Participating healthcare professionals were encouraged to take a more personal than professional role.  PS as an educational approach can improve the collaboration between professionals and users.  The collaboration will change the roles and responsibilities of professionals and users and promote user-centric care.  A positive effect of the PS is improved collaboration between psychiatry units -also promoted by staff with user experience.  Summary:  PS is a patient-centric activity which helps to normalise mental illness and reduce stigma. This educational activity improves collaboration between staff and users, requiring changes in roles and responsibilities, and between psychiatry units. | Perceived value:  Users  (Six focus group interviews and eight individual interviews - findings were in line with earlier participant feedback). The course as a *social forum* was especially appreciated, participants mentioned its *“good atmosphere”* and that *lasting contacts were created, including those with professional staff*. Participants learned *useful methods* to set limits and learned about their *legal rights*, one example being the right to an individual work plan.  Clinical staff views:  The *informants’ knowledge* of the activities at Sagatun Recovery and particularly the Self-Empowerment Course and its perceived value were *relatively scarce*. Nevertheless, three of the informants had been on regular visits to the centre and they had worked actively to introduce Sagatun Recovery as a low-threshold service to patients they serve. None of the informants in our study had inside information *of the course*, but all of them *knew the existence of the course* and knew of people having attended it. They all had the impression *that this course represents a useful supplement to the existing public mental health services*, both psychiatric specialist care and services provided locally by the municipalities. They were *overall positive to this way of doing education* and supposed that it gave the participants useful *tools in order to manage both their illness and life in general*.  Summary:  *Valuable to users* were:  The social forum and its good atmosphere.  Lasting contacts with peers and professional staff.  Useful methods to set limits in life.  Knowledge about legal rights.  *Professional staff observations:*  Limited knowledge of the activities at Sagatun, particularly the Self-Empowerment Course.  Some had made regular visits to the centre and they had worked actively to introduce Sagatun Recovery.  None had inside information of the course  All knew the existence of the course and knew of people having attended it.  The view was that this course represents a useful supplement to the existing public mental health services.  This kind of educational activity is valuable and provides useful tools which help users to manage their illness and life in general.  Summary:  Staff have limited insights into the Self-empowerment course but knew patients who had attended. The course is a useful supplement to mental health services and provides participants with useful self-management tools. |
| Other | No impact on clinical practice, seen as a supplement to care. | No impact on clinical practice, seen as a supplement to care. |
